# Vitamin D Status and Gestational Diabetes in Russian Pregnant Women in the Period between 2012 and 2021: A Nested Case–Control Study

**DOI:** 10.3390/nu14102157

**Published:** 2022-05-22

**Authors:** Aleksandra S. Tkachuk, Elena A. Vasukova, Anna D. Anopova, Tatiana L. Karonova, Evgenii A. Pustozerov, Yana A. Teplova, Angelina I. Eriskovskaya, Artem O. Isakov, Elena Y. Vasilieva, Maria A. Kokina, Irina Y. Zazerskaya, Tatiana M. Pervunina, Elena N. Grineva, Polina V. Popova

**Affiliations:** 1 Institute of Endocrinology, Almazov National Medical Research Centre, 194156 Saint Petersburg, Russia; aleksandra.tkachuk.1988@mail.ru (A.S.T.); elenavasukova2@gmail.com (E.A.V.); karonova@mail.ru (T.L.K.); pustozerov.e@gmail.com (E.A.P.); yanabolotko@gmail.com (Y.A.T.); grineva_e@mail.ru (E.N.G.); 2 World-Class Research Center for Personalized Medicine, Almazov National Medical Research Centre, 194156 Saint Petersburg, Russia; anopova.ann@gmail.com (A.D.A.); yeriskovskaya@gmail.com (A.I.E.); amedi.ioakim@gmail.com (A.O.I.); mapillika@yandex.ru (M.A.K.); 3 Department of Biomedical Engineering, Saint Petersburg State Electrotechnical University, 197341 Saint Petersburg, Russia; 4 Central Clinical Diagnostic Laboratory, Almazov National Medical Research Centre, 194156 Saint Petersburg, Russia; elena-almazlab@yandex.ru; 5 Department of Obstetrics and Gynecology, Almazov National Medical Research Centre, 194156 Saint Petersburg, Russia; zazerskaya@almazovcentre.ru; 6 Institute of Perinatology and Pediatrics, Almazov National Medical Research Centre, 197341 Saint Petersburg, Russia; pervunina_tm@almazovcentre.ru; 7 Department of Internal Diseases and Endocrinology, St. Petersburg Pavlov State Medical University, 197022 Saint Petersburg, Russia

**Keywords:** gestational diabetes mellitus, 25(OH)D level, vitamin D deficiency, vitamin D supplementation

## Abstract

Several meta-analyses found an association between low maternal serum 25-hydroxyvitamin D (25(OH)D) level and gestational diabetes mellitus (GDM). However, some of them reported significant heterogeneity. We examined the association of serum 25(OH)D concentration measured in the first and in the second halves of pregnancy with the development of GDM in Russian women surveyed in the periods of 2012–2014 and 2018–2021. We conducted a case–control study (including 318 pregnant women) nested on two previous studies. In 2012–2014, a total of 214 women (83 GDM and 131 controls) were enrolled before 15 weeks of gestation and maternal serum 25(OH)D concentrations were measured twice: at 8th–14th week of gestation and simultaneously with two-hour 75 g oral glucose tolerance test (OGTT) at 24th–32nd week of gestation. In the period of 2018–2021, 104 women (56 GDM and 48 controls) were included after OGTT and 25(OH)D concentrations were measured at 24th–32nd week of gestation. Median 25(OH)D levels were 20.0 [15.1–25.7] vs. 20.5 [14.5–27.5] ng/mL (*p* = 0.565) in GDM and control group in the first half of pregnancy and 25.3 [19.8–33.0] vs. 26.7 [20.8–36.8] ng/mL (*p* = 0.471) in the second half of pregnancy, respectively. The prevalence rates for vitamin D deficiency (25(OH)D levels < 20 ng/mL) were 49.4% and 45.8% (*p* = 0.608) in the first half of pregnancy and 26.2% vs. 22.1% (*p* = 0.516) in the second half of pregnancy in women who developed GDM and in women without GDM, respectively. The frequency of vitamin D supplements intake during pregnancy increased in 2018–2021 compared to 2012–2014 (*p* = 0.001). However, the third trimester 25(OH)D levels and prevalence of vitamin D deficiency (25.5 vs. 23.1, *p* = 0.744) did not differ in women examined in the periods of 2012–2014 and 2018–2021. To conclude, there was no association between gestational diabetes risk and maternal 25(OH)D measured both in the first and in the second halves of pregnancy. The increased prevalence of vitamin D supplements intake during pregnancy by 2018–2021 did not lead to higher levels of 25(OH)D.

## 1. Introduction

The increasing prevalence of overweight and obesity in the general population also contributes to the continuing increase in the prevalence of GDM, which currently complicates approximately 13–14% of pregnancies [1]. GDM is accompanied by an increased risk of adverse health outcomes for both mother and offspring [2,3]. Although many risk factors for GDM are well known, including advanced maternal age, obesity, family history of diabetes, and ethnicity [4,5], the mechanism underlying the role of these factors in GDM predisposition remains an area of active research [6]. In recent years, multiple new potential risk factors have emerged, including vitamin D deficiency [7].

Growing number of studies have shown the association of vitamin D deficiency with GDM risk [8,9,10]. However, some studies did not confirm this association [11,12,13] and even revealed a positive association of 25-hydroxyvitamin D (25(OH)D) level with glucose concentration during pregnancy [14]. Several meta-analyses found an association between low maternal serum 25(OH)D levels and GDM [15,16,17]. However, some of them reported significant heterogeneity [18,19], which may be due to different criteria of GDM [20], different gestational ages at the time of vitamin D status measurement and the diversity of study populations in terms of ethnicity.

Overall, these meta-analyses have shown an association of vitamin D insufficiency with the risk of developing gestational diabetes [21]. However, it should be noted that these meta-analyses are based on observational studies and their quality is limited by the mixing of diverse study populations, different laboratory techniques and the time of 25(OH)D measurement. Most importantly, the presence of a relationship does not prove a causal relationship.

Recent systematic reviews of randomized controlled trials concerning the effect of vitamin D supplements use on GDM risk have shown conflicting results. The authors of two meta-analyses of randomized controlled trials have concluded that vitamin D supplementation did not influence the risk of GDM [22,23]. However, a systematic review by Palacios C. et al. (2019) on various aspects of vitamin D supplementation in pregnancy concluded that vitamin D intake in pregnancy possibly reduces the risk of GDM (OR 0.51, 95% CI 0.27–0.97) based on the inclusion of four RCTs (446 women) with moderate strength of evidence [24]. Similar results were obtained by Griffith JG et al. summarizing the evidence from Cochrane systematic reviews [25]. The authors concluded that supplementation with vitamin D was of possible benefit in reducing the risk of GDM, which require further high-quality evidence.

Vitamin D supplementation also poses a potentially attractive target in the treatment of GDM [26]. Usually, the preference among treatment options for GDM is given to insulins [27]. Some medical societies recommend sulfonylurea and metformin for the management of GDM [28], but safety concerns preclude recommendation of oral agents during pregnancy by other organizations [29]. Among non-pharmacological agents, myo-inositol supplementation is widely studied for GDM prevention and reduction of GDM-related complications [30]. Several randomized controlled trials and a subsequent meta-analysis have shown benefits of vitamin D supplementation on glycemic control in women with GDM [26]. However, as the authors warn, “due to the limited number of studies in the meta-analysis, the conclusion should be interpreted with caution” [26].

The implications of vitamin D on other reproductive disorders (premature ovarian failure, endometriosis, polycystic ovary syndrome, gynecological cancer and uterine fibroids) are also widely discussed [31]. Although associations of serum 25(OH)D levels with several gynecological diseases affecting female fertility as well as with IVF and pregnancy outcomes have been described, no conclusive results have been found after vitamin D supplementation [31,32].

A plausible explanation for the conflicting results is the use of different doses of vit D and higher effect of higher doses to reduce the risks. However, to date, the literature does not support the routine prescription of high doses of vitamin D during pregnancy to prevent or treat GDM and adverse pregnancy outcomes.

The Endocrine Society suggests that pregnant and lactating women require at least 600 IU/d of vitamin D and recognize that at least 1500–2000 IU/d of vitamin D may be needed to maintain a 25(OH)D level above 30 ng/mL [33]. A Russian guideline with recommended vitamin D intake of at least 800–1200 IU/d during pregnancy was for the first time published in 2016 and has drawn attention to the need for vitamin D supplements intake during pregnancy [34].

We aimed to examine the association of serum 25(OH)D concentration measured in the first and/or in the second halves of pregnancy with the development of GDM in Russian women surveyed before and after the implementation of the guideline on vitamin D deficiency prevention.

## 2. Materials and Methods

### 2.1. Study Population

For the purpose of this nested case–control study we selected participants from two studies performed in the Almazov National Medical Research Centre (NMRC):

1. The prospective observational study of early markers of GDM conducted from January 2012 to December 2014;
2. GEM-GDM randomized controlled trial (Genetic and Epigenetic Mechanisms of Developing Gestational Diabetes Mellitus and Its Effects on the Fetus) performed from July 2015 to July 2021 [35].

The prospective observational study population included 1050 pregnant women (age 18–45 years) who were screened for risk factors of GDM in the first trimester of pregnancy. The women were under observation at ObGyn clinics № 22 (including four ObGyn clinics) in terms of routine medical care. Study participants were invited to Almazov Centre for analyses twice during pregnancy. In the first trimester fasting blood samples were collected from all the participants. Serum and plasma were stored at −80 °C for further analyses. Between weeks 24 and 32, the women were invited for a 2 h oral glucose tolerance test (OGTT) with 75 g glucose and again serum and plasma were stored at −80 °C for further analyses.

The diagnosis of GDM was made using a single-step 75-g OGTT according to the recommendation of the International Association of Diabetes and Pregnancy Study Groups (IADPSG) [36]. Appropriate medical care for women diagnosed with GDM was provided in the Almazov Centre.

The women with GDM were selected for the present study if their serum samples collected at the 9–13th week of gestation were available. In total, 83 women with GDM fulfilled these criteria.

The controls were randomly selected from women without GDM in the cohort. The women with fasting glucose levels >7.0 mmol/L in the first trimester, a history of pre-gestational diabetes and other diseases affecting carbohydrate metabolism were excluded.

The design of the GEM-GDM study is reported elsewhere (ClinicalTrials.gov identifier: NCT03610178) [35]. In brief, the participants of the study were pregnant women with and without GDM as confirmed by OGGT data (age 18–45 years). The GEM-GDM study aimed to compare pregnancy outcomes in women with GDM who were randomly assigned into 2 groups with different target glycemic levels (strict and liberal), but for the purpose of the study reported here, we used the serum samples obtained before the randomization. Thus, the two groups were merged in one GDM group. GDM was diagnosed according to the recommendations of the IADPSG [36]. Participants were included at a gestational age of <32 weeks. If normal glucose tolerance was confirmed by OGTT at 24–31 weeks of gestation, a woman was included in the control group. Like in the observational study, we excluded women with pre-existing diabetes and other medical conditions affecting glucose metabolism. As we aimed to assess the effect of the implementation of Russian guidelines on vitamin D deficiency prevention, we included in the present study the women who participated in the GEM-GDM study in 2018–2021.

All subjects gave their informed consent for inclusion before they participated in the study. Both studies were conducted in accordance with the Declaration of Helsinki, and their protocols were approved by the Ethics Committee of the Almazov NMRC (Protocol 4A, 16 January 2012 and protocol 119, 13 July 2015).

The data concerning medical history, including arterial hypertension, impaired glucose tolerance (IGT), GDM, polycystic ovary syndrome (PCOS), family history of diabetes, pre-gestational weight and parity, were collected from medical charts. The calculation of pre-gestational body mass index (BMI) was performed by dividing weight (in kilograms) by the square of height (in meters).

The data concerning the intake of vitamin D containing supplements were collected using self-reported questionnaires administered by the time of blood sampling in the third trimester. The questionnaire included two questions: Vitamin D supplements intake in the first trimester (yes/no) and Vitamin D supplements intake during pregnancy (No/<1 month/1–3 months/>3 months).

For the women participating in 2018–2021 the doses of cholecalciferol in their supplements were available in the medical charts.

### 2.2. Assessment of Biochemical Parameters

Blood samples were drawn from the participants of the prospective study (2012–2014) twice: at the time of recruitment in the first trimester and at the time OGTT at 24th–31st week of gestation, then frozen at −80 °C, and analyzed in batches. The participants of the GEM-GDM study provided their first blood samples at the 24th–31st week of gestation before the intervention. Thus, their levels of 25(OH)D were analyzed only once in the third trimester.

Serum 25(OH)D level was analyzed by chemiluminescence immunoassay (Abbott Architect c8000, Chicago, IL, USA, inter-assay CV of 2.15–2.63%, intra-assay CV ranged from 1.60 to 5.92%). Normal vitamin D status was defined as 25(OH)D ≥30 ng/mL (≥75 nmol/L) and the following thresholds were used for insufficiency, ≥20 and <30 ng/mL (≥50 and <75 nmol/L); for deficiency, <20 ng/mL (<50 nmol/L), and for severe vitamin D deficiency, less than 10 ng/mL (<25 nmol/L) in accordance with Russian and international guidelines [33,34]. The reference interval for serum 25(OH)D level was 3.4–155.9 ng/mL.

At the 24th–31st week of gestation, the 2 h oral glucose tolerance test (OGTT) with 75 g glucose was performed. Glucose oxidase method was used to determine glucose concentration in fresh plasma samples.

### 2.3. Statistical Analysis

The sample size was calculated using Select Statistical Services (select-statistics.co.uk). A total of 99 participants in each group are required to have an 80% chance of detecting a difference of 10 in the level of 25(OH)D with the population variance of 625 (based on our previous results) and with 95% confidence level.

Statistical analysis was performed using SPSS 26.0 software (SPSS Inc., Chicago, IL, USA).

Quantitative data were assessed for normality using the Kolmogorov–Smirnov test.

In the case of normal distribution, the data were described as mean and average. In the absence of a normal distribution, quantitative data were described using median (Me) and lower and upper quartiles (Q1 to Q3).

Categorical data were described as absolute values and percentages. Differences in the quantitative characteristics of the groups were assessed with Mann–Whitney U-test. Percentages were compared by Pearson’s chi-square test (for expected values greater than 10) and Fisher’s exact test (for expected values less than 10). To identify the contribution of 25(OH)D to the risk of GDM, binary logistic regression was used.

## 3. Results

### 3.1. Characteristics and Vitamin D Status of Women with and without GDM

In total, 318 pregnant women (138 GDM cases and 180 controls) were included. The characteristics of the participants are presented in Table 1.

The women suffering from GDM were older, had higher BMI before pregnancy, more often had chronic arterial hypertension and had higher levels of plasma glucose in OGTT. There was no difference in terms of the history of IGT, GDM, family history of Type 2 diabetes mellitus and parity.

There was no difference in the 25(OH)D levels in the first and in the third trimester of pregnancy (*p* = 0.565 and 0.471, respectively).

The prevalence of vitamin D deficiency (<20 ng/mL) was high in the first trimester and did not differ between the groups (49.4% and 45.8%, *p* = 0.674). It decreased by the third trimester and tended to be higher in the GDM group, but the difference was not significant (26.2% and 22.1%, *p* = 0.516).

The proportion of women who reported intake of Vitamin D supplements in the first trimester and during pregnancy was comparable between the groups (*p* = 0.124 and 0.134, respectively).

However, among women who participated in 2018–2021 those with GDM took higher doses of vitamin D in the third trimester compared with the control group (*p* < 0.001).

The multivariate analysis (Table 2) showed that there was no association between serum 25(OH)D concentration and GDM risk (adjusted OR = 1.03, *p* = 0.244 for the first-trimester level and adjusted OR = 1.00, *p* = 0.486 for the third-trimester level). There was also no association between GDM and vitamin D insufficiency or vitamin D deficiency (Table 2).

### 3.2. Vitamin D Status in the Third Trimester and Related Characteristics of Pregnant Women Participating in the Period 2012–2014 and 2018–2021

In order to assess the influence of implementation of Russian guidelines [34] on vitamin D deficiency prevention, we compared 25(OH)D levels in the third trimester, reported intakes of vitamin D supplements and related characteristics of pregnant women recruited in the period 2012–2014 and those included in 2018–2021 (Table 3).

The women participating in 2018–2021 were older and more often were multiparae compared to the participants of the observational study in 2012–2014. There was no difference in pre-pregnancy BMI, the levels of plasma glucose in OGTT and other related characteristics between the groups surveyed in 2012–2014 and 2018–2021.

**Figure 1:**
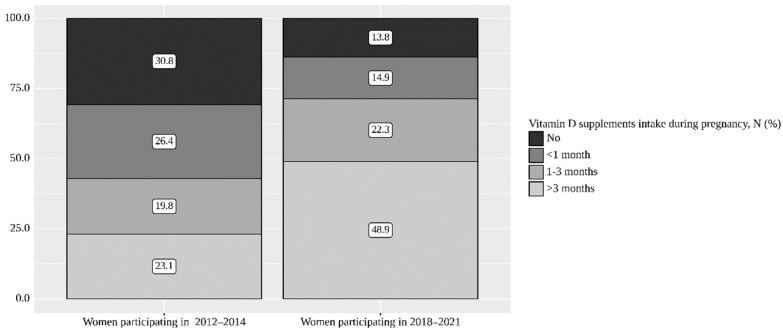
Prevalence of vitamin D supplement intake during pregnancy in 2011–2014 and 2018–2021.

A higher proportion of women reported Vit D supplements intake during pregnancy for three and more months (48.9% vs. 23.1%), while the proportion of women without Vitamin D supplements intake decreased from 30.8% in 2012–2014 to 13.8% in 2018–2021 (*p* < 0.001) (Table 3 and Figure 1).

However, in spite of this improvement, there was no difference in the third trimester 25(OH)D levels (*p* = 0.827), as well as in the prevalence of vitamin D deficiency (<20 ng/mL) (25.5 vs. 23.1, *p* = 0.744) between the two cohorts.

Women who participated in the study in 2018–2021 took supplements containing cholecalciferol at a median dose of 400 [400–2400] IU, with a minimum dose of 250 IU and a maximum of 6000 IU.

### 3.3. 25OH-Vitamin D Levels Change during Pregnancy

The levels of 25(OH)D were available in both time points (in the first trimester and at 24th–31st week of gestation) in 91 women (49 GDM and 42 controls) who were recruited in 2011–2014.

There was a statistically significant increase in 25(OH)D levels by gestational age 24–31 weeks both in the women with GDM and the control group (*p* < 0.001 in both groups) (Table 4). The increase of 25(OH)D level in the third trimester compared to the first trimester was observed in 81.6% of women with GDM and in 64.3% of women without GDM. There was a positive correlation between 25(OH)D levels measured in the first and in the third trimesters (r = 0.436, *p* = 0.002 in the GDM group and r = 0.535, *p* < 0.001 in the control group).

## 4. Discussion

Our nested case–control study did not confirm the association of 25(OH)D levels recorded in the first and in the third trimesters of pregnancy with GDM diagnosed according to IADPSG criteria.

In multiple previous studies, lower levels of serum 25(OH)D were associated with higher GDM risk and this association was confirmed by some meta-analyses [15,16,17,18,19].

Nevertheless, several case–control studies did not find a relationship between 25(OH)D levels/vitamin D deficiency and the development of GDM after adjusting for possible confounders, including recent large studies published after the aforementioned meta-analyses were performed [13,37,38,39].

A study from Taiwan has even shown opposite results with the higher levels of 25(OH)D in women with abnormal GCT compared to women with normal glucose tolerance during pregnancy [14].

Another study from Taiwan found a nonlinear association between serum 25(OH)D concentration during pregnancy and the risk of GDM. In this study, low and high levels of 25(OH)D were associated with the increased risk of GDM, whereas moderate levels were associated with lower GDM risk [40].

The inconsistency of the evidence concerning the association of 25(OH)D levels with GDM makes us believe that such an association, even if confirmed on a larger sample size, may be of little clinical significance. This premise is confirmed by randomized studies of vitamin D supplementation during pregnancy, which failed to show clear benefits in terms of GDM risk reduction [22,23,24,25,41,42].

However, the elusiveness of the benefits of vitamin D for GDM prevention does not diminish the importance of adequate levels of vitamin D for the prevention of other health-related problems and complications of pregnancy [33,34].

Another goal of our study was to assess the effect of the implementation of Russian guidelines [34] concerning prevention of vitamin D deficiency during pregnancy.

We observed no difference in the third trimester 25(OH)D levels between the women participating in the period 2012–2014 and 2018–2021 despite the increase of the proportion of those taking vitamin D supplements during pregnancy in 2018–2021. It may be due to the low doses of vitamin D taken by the participants (median dose of 400 IU/day).

There is a discrepancy between the recommendations of the Russian association of endocrinologists and the recommendations of the Russian association of obstetricians and gynecologists concerning vitamin D supplementation during pregnancy. The Russian association of endocrinologists recommends 800–1200 ME/day for all pregnant women [34], while the Russian association of obstetricians and gynecologists recommends 400 ME/day only for women at risk for vitamin D deficiency. It was shown in several studies that the doses of vitamin D required to reach sufficient serum 25(OH)D levels are much higher than 400 ME/day [33,43].

The prevalence of vitamin D deficiency in the first half of pregnancy in our study (49.4% and 45.8% in women who developed GDM and in women without GDM, respectively) was in line with the data from the non-pregnant residents of North-West region of Russia (45.7%) [44]. The prevalence of vitamin D deficiency decreased by the second half of pregnancy in both groups (26.2% and 22.1%, *p* = 0.516).

The absence of differences in vitamin D status between women with GDM and the control group, despite higher doses of vitamin D intake in the GDM group, may be due to the fact that they started taking vitamin D a short time (less than 2 weeks) before blood sampling and this did not significantly change the vitamin D level. Usually, obstetricians prescribe low doses of vitamin D in multivitamins to all pregnant women, but endocrinologists prescribe vitamin D in higher doses after diagnosing GDM. In our study, some women were tested for vitamin D during OGTT (if they were screened at the Almazov Center), but in 2018–2021, most of the participants were tested within 2 weeks after GDM was diagnosed.

Among limitations, it should be noted that this is not a population-based study and some confounders associated with the observational study selection may affect the results (for example, different age of women participating in the periods of 2012–2014 and 2018–2021). One more limitation is that we do not have data on each participant’s doses of vitamin D supplements in 2012–2014. However, we suppose that the doses were low (250–400 IU/day) basing on the absence of national guidelines recommending higher doses at that time. Another limitation is a relatively small sample size which could preclude detection of the association of 25(OH)D levels with GDM. However, we believe that such an association may be of little clinical significance even if confirmed on larger sample size.

## 5. Conclusions

Our study did not confirm the association of 25(OH)D levels in the first and second halves of pregnancy with GDM risk. However, as achievement of adequate levels of 25(OH)D is recommended for other health benefits, we also aimed to evaluate the effect of the implementation of Russian guidelines [34] concerning the prevention of vitamin D deficiency during pregnancy. A higher proportion of women surveyed after implementation of the guideline reported vitamin D supplements intake during pregnancy, but it did not lead to higher serum 25(OH)D levels. The prevalence of vitamin D deficiency and insufficiency remained high in women included in 2018–2021, presumably due to the insufficient doses of cholecalciferol.

## Figures and Tables

**Table 1:** Vitamin D status and characteristics of women with and without GDM in the whole study period.

	Women with GDM *N* = 138	Women without GDM *N* = 180	*p*
Age	31 [28.0–34.0]	29 [26.0–33.0]	0.005
Pre-gestational BMI	23.4 [21.4–27.2]	22.4 [20.4–24.9]	0.010
Family history of diabetes, ***N*** (%)	56 (40.0)	63 (34.2)	0.287
History of arterial hypertension, ***N*** (%)	19 (13.6)	12 (6.5)	0.033
History of GDM, ***N*** (%)	6 (7.2)	2 (2.0)	0.144
History of IGT, ***N*** (%)	3 (2.1)	3 (1.6)	1.000
PCOS, ***N*** (%)	8 (5.7)	11 (6.0)	0.911
Parity:			
Nulliparae, ***N*** (%)	91 (65.0)	111 (60.3)	0.390
Multiparae, ***N*** (%)	49 (35.0)	73 (39.7)	
Fasting plasma glucose (mmol/L)	5.2 [4.7–5.4]	4.3 [4.0–4.5]	<0.001
1 h postload glucose (mmol/L)	9.0 [7.6–10.1]	6.7 [5.7–7.6]	<0.001
2 h postload glucose (mmol/L)	7.6 [6.0–8.8]	6.0 [5.1–6.5]	<0.001
Vitamin D supplements intake in the first trimester, ***N*** (%)	37 (29.1)	34 (21.2)	0.124
Vitamin D supplements intake during pregnancy, ***N*** (%)			
No	30 (23.6)	53 (33.1)	0.134
<1 month	28 (22.0)	42 (26.2)	
1–3 months	27 (21.3)	25 (15.6)	
>3 months	42 (33.1)	40 (25.0)	
Doses of cholecalciferol, IU ***	400 [400–2400]	400 [400–400]	<0.001
Gestational age at the time of 25(OH)D analysis in the 1st trimester, weeks *	11 [8–13]	10 [8–12]	0.028
First trimester 25(OH)D level, ng/mL *	20.0 [15.1–25.7]	20.5 [14.4–27.5]	0.565
Severe vitamin D deficiency in the first trimester (<10 ng/mL), ***N*** (%) *	5 (6.0)	3 (2.3)	0.266
Vitamin D deficiency in the first trimester (>10 and <20 ng/mL), ***N*** (%) *	36 (43.4)	57 (43.5)	0.608
Vitamin D insufficiency in the first trimester (>20 and <30 ng/mL), ***N*** (%) *	32 (38.6)	46 (35.1)	0.610
Adequate level of 25(OH)D in the first trimester, ***N*** (%) *	10 (12.0)	25 (19.1)	0.175
Excessive level of 25(OH)D in the first trimester (>100 ng/mL)	0	0	
Gestational age at the time of 25(OH)D analysis in the 3rd trimester, weeks **	26.5 [25.0–29.0]	26.0 [25.0–28.2]	0.438
Third trimester 25(OH)D level, ng/mL **	25.3 [19.7–32.9]	26.7 [20.7–36.7]	0.471
Severe vitamin D deficiency in the third trimester (<10 ng/mL), ***N*** (%)	2 (1.9)	6 (6.3)	0.151
Vitamin D deficiency in the third trimester (>10 and <20 ng/mL), ***N*** (%)	26 (24.3)	15 (15.8)	0.133
Vitamin D insufficiency in the third trimester (>20 and <30 ng/mL), ***N*** (%)	42 (39.2)	40 (42.1)	0.680
Adequate level of 25(OH)D in the third trimester, ***N*** (%)	37 (34.6)	34 (35.8)	0.857
Excessive level of 25(OH)D in the third trimester (>100 ng/mL)	0	0	

* 25(OH)D level was determined in 131 women without GDM and in 83 women with GDM in the first trimester; ** 25(OH)D level was determined in 95 women in the control group and 107 women with GDM in the third trimester; *** Doses of cholecalciferol intake were available in 48 women without GDM and 55 women with GDM who participated in the 2018–2021 study.

**Table 2:** Association between vitamin D levels during pregnancy and gestational diabetes mellitus risk.

Vitamin D Levels	Crude Model			Adjusted Model *		
	OR	95% CI	*p*	OR	95% CI	*p*
First trimester 25(OH)D concentration	0.98	0.95–1.02	0.315	1.03	0.95–1.06	0.244
First-trimester vitamin D insufficiency						
≥30 ng/mL	1.00		0.168	1.00		0.222
<30 ng/mL	1.72	0.78–3.80		1.39	0.38–5.07	
First-trimester vitamin D deficiency						
≥20 ng/mL	1.00		0.608	1.00		0.218
<20 ng/mL	1.16	0.67–2.00		1.32	0.48–3.61	
Third trimester 25(OH)D concentration	0.99	0.97–1.01	0.443	1.00	0.96–1.03	0.486
Third-trimester vitamin D insufficiency						
≥30 ng/mL	1.00		0.857	1.00		0.489
<30 ng/mL	1.05	0.59–1.88		1.17	0.50–2.41	
Third-trimester vitamin D deficiency						
≥20 ng/mL	1.00		0.501	1.00		0.494
<20 ng/mL	1.25	0.65–2.39		1.08	0.44–2.66	

* Logistic regression analyses adjusted for pre-gestational BMI, age, parity, GDM in history, impaired glucose tolerance, arterial hypertension and family history of diabetes.

**Table 3:** Vitamin D status in the third trimester and related characteristics of pregnant women participating in the period 2012–2014 and 2018–2021.

	Women Participating in 2012–2014 *N* = 98 *	Women Participating in 2018–2021 *N* = 104 *	*p*
Age	30 [27–33]	31 [28–35]	0.008
Pre-gestational BMI	23.3 [20.9–26.9]	22.2 [20.4–24.8]	0.098
*N* (%) of women with GDM	51 (52.0%)	56 (53.8%)	0.888
Family history of diabetes, *N* (%)	40 (40.8)	40 (38.5)	0.732
History of arterial hypertension, *N* (%)	10 (10.2)	5 (4.8)	0.183
History of GDM, *N* (%)	1 (1.0)	6 (5.8)	1.000
History of IGT, *N* (%)	4 (4.1)	0 (0.0)	0.054
PCOS, *N* (%)	4 (4.1)	7 (6.7)	0.539
Parity:			
Nulliparae, *N* (%)	70 (71.4)	62 (59.6)	0.078
Multiparae, *N* (%)	28 (28.6)	42 (40.4)	
Fasting plasma glucose (mmol/L)	4.7 [4.4–5.3]	4.6 [4.2–5.1]	0.072
1 h postload glucose (mmol/L)	7.8 ± 1.9	7.7 ± 1.7	0.457
2 h postload glucose (mmol/L)	6.4 [5.5–8.2]	6.5 [5.5–7.7]	0.777
Vitamin D supplements intake in the first trimester, *N* (%)	21 (23.1)	28 (29.8)	0.301
Vitamin D supplements intake during pregnancy, *N* (%)			
No	28 (30.8%)	13 (13.8%)	<0.001
<1 month	24 (26.4%)	14 (14.9%)	
1–3 months	18 (19.8%)	21 (22.3%)	
>3 months	21 (23.1%)	46 (48.9%)	
Doses of cholecalciferol, ME **	-	400 [400–2000]	-
Third trimester 25(OH)D level, ng/mL	25.6 [20.0–32.8]	26.3 [20.3–35.0]	0.827
Severe vitamin D deficiency in the third trimester (<10 ng/mL), *N* (%)	3 (3.1)	5 (4.8)	0.722
Vitamin D deficiency in the third trimester (>10 and <20 ng/mL), *N* (%)	22 (22.4)	19 (18.3)	0.460
Vitamin D insufficiency in the third trimester (>20 and <30 ng/mL), *N* (%)	39 (39.8)	43 (41.3)	0.823
Adequate level of 25(OH)D, *N* (%)	34 (34.7)	37 (35.6)	0.895
Excessive (>100 ng/mL)	0	0	

* Only women with available data on the third trimester Vitamin D levels were included in this table. ** 89 women that took part in the 2018–2021 study.

**Table 4:** Serum 25(OH)D levels in women with and without GDM in 2012–2014 depending on gestational age.

	Serum 25(OH)D Levels, Me [Q1–Q3], ng/mL		***p***
	1st Trimester	3rd Trimester	
Women with GDM, *N*= 49	19.1 [14.0–26.5]	25.2 [19.3–32.3]	<0.001
Women without GDM, *N* = 42	22.8 [13.5–30.2]	26.8 [21.5–35.1]	<0.001

## Data Availability

The data presented in this study are available upon request from the corresponding author.
